# The effect of the facilitated tucking position in reducing vaccination-induced pain in newborns

**DOI:** 10.1186/s13052-015-0168-9

**Published:** 2015-08-21

**Authors:** Sibel Kucukoglu, Sirin Kurt, Aynur Aytekin

**Affiliations:** Department of Child Health Nursing, Faculty of Health Sciences, Atatürk University, 25240 Erzurum, Turkey; Neonatal Intensive Care Unit, Hospital of Istanbul Medical Faculty, İstanbul University, 34093 İstanbul, Turkey

## Abstract

**Background:**

This study was conducted to evaluate the pain perceptions of newborns during the hepatitis B (HBV) vaccinations performed in the facilitated tucking position and the classical holding position, respectively.

**Methods:**

The randomized controlled experimental study was conducted between 1 September 2014 and 30 December 2014 at the neonatal intensive care unit of a Turkish university hospital. One group of infants was held in the facilitated tucking position (the treatment group; *n* = 30) during HBV vaccination; infants in the other group were held in the classical holding position (the control group; *n* = 30) during HBV vaccination. The Neonatal Infant Pain Scale (NIPS) scores of the infants in the treatment and control groups were compared during procedure. Also, the infants’ physiological parameters were compared before, during, and after the procedure. Descriptive statistics, a chi-square test, and an independent samples *t*-test were used to assess the data.

**Results:**

The mean pain scores of infants vaccinated in the facilitated tucking position (2.83 ± 1.18) were significantly statistically lower than the scores of infants vaccinated in the classical holding position (6.47 ± 1.07) (*p* < 0.05).

**Conclusions:**

The pain perceptions of newborns held in the facilitated tucking position during HBV vaccination were lower. The facilitated tucking position, a non-pharmacological method, is recommended as an effective and useful method for reducing pain during the procedure.

## Introduction

Pain in newborns is a complicated, individualized, subjective, and universal finding [1]. The most common painful procedures performed during infancy are routine injections without pain management [1, 2]. Vaccinations are first administered when babies are very young [1]. Studies have shown that uncontrolled pain experienced during the early stages of life has negative and long-term side effects, such as distress, and that such pain negatively affects the development of the central nervous system [3–6].

The most important problem encountered while evaluating pain in newborns is the inability of babies to express pain verbally. Attention should be paid to non-verbal indications during communication established with infants. Physiological parameters, behavioral methods, and stress hormones have been evaluated to define the pain felt by newborns [7]. Pain experienced by newborns affects the heart rate, respiration rate, blood pressure, and tissue oxygenation, potentially causing these parameters to decrease or increase [8].

Pharmacological methods used to relieve pain in newborns are reported to have side effects such as respiratory depression, apnea, bradycardia, hypotension, desaturation, partial airway obstruction, and hypersalivation [9, 10]. Non-pharmacological and pharmacological methods are considered for pain relief by health stuff. These methods are valuable alternatives for pain control during brief invasive procedures performed on newborns [11, 12]. One method, the facilitated tucking position, is defined as “a sub-form of method of nesting the baby and the procedure of bringing the body to middle or even close position by holding the upper and lower extremities of the baby in flexion with hands.” An infant can be held in the lateral, supine, or prone position while this method is performed [12, 13]. It has been reported to prompt infants’ own regulatory systems, prevent painful stimulants coming from the outside world, and reduce the pain felt by the infant by enabling heat and touching stimuli [14–16]. In addition, this method stabilizes infants’ physiological parameters and helps them gain a feeling of safety based on the position, supports their motor development, and preserves their energy [15].

Although many studies are reported on procedural pain control and assessment in the infants [13, 15, 17–24], there are no studies on facilitated tucking position to alleviate the pain associated with the vaccination. Recent studies have focused on pain physiology, pain assessment, and pharmacological interventions [4, 25]. The primary responsibility of a nurse is to ensure that pain-relieving methods are performed before and after a procedure during routine practices in addition to determining and relieving the pain. After an accurate assessment, the pain felt by a neonate can be managed through effective care provided by family-centered and individualized pharmacological and non-pharmacological methods [26].

The purpose of this study was to evaluate the pain perceptions of newborns during HBV vaccinations performed in the facilitated tucking position and the classical holding position, respectively.

## Materials and methods

### Study design

This randomized controlled experimental study was conducted at the neonatal intensive care unit of a Turkish university hospital. The study population consisted of newborns in the neonatal intensive care unit between 1 September 2014 and 30 December 2014 who met the inclusion criteria. The study was conducted on the entire population; no sample group was selected.

#### Inclusion criteria

Infants with gestational ages of 37 weeks and over, birth weights of more than 2500 g, stable medical conditions, and mothers in the early postpartum period who volunteered to participate were included in the study.

#### Exclusion criteria

Newborns who had congenital anomalies, required positive pressure ventilation support, and received pain-relieving and sedative treatment were not included in the study.

**Fig. 1:**
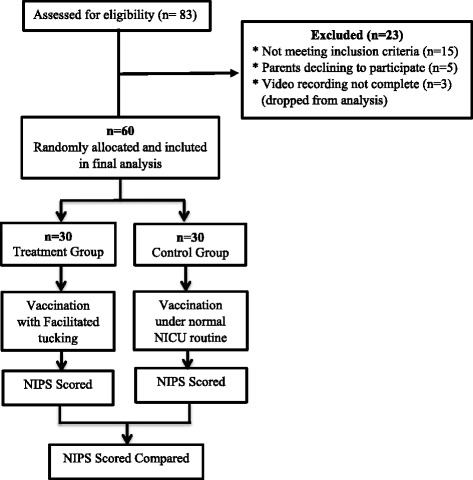
Flow of study

The study population consisted of 60 infants, who were divided into the facilitated tucking position group (the treatment group; *n* = 30) and the classical holding position group (the control group; *n* = 30). The sample size was calculated for the independent samples *t*-test with 80 % power, significance at the 0.05 level, and standard deviation of 2.16 [27]. The Power and Sample Size program software determined that a sample of 30 subjects in the treatment and control groups would be needed to reject the null hypothesis. The infants were randomized to either the treatment group or the control group in restricted block randomization to ensure a ratio of 1:1. Small blocks cause a degree of predictability; therefore, a block size of 10 was used. Cards prepared with assignments were kept in sealed envelopes and shuffled to produce a form of random assignment [28] (Fig. 1).

### Data collection instruments

The data were collected with the Personal Information Form, Intervention Follow-up Form, and Neonatal Infant Pain Scale by the researcher. The Personal Information Form prepared by the researchers was used to collect descriptive characteristics of the newborn (gender, gestational age, weight, height, delivery method, number of children previously delivered by the baby’s mother, etc.). The Intervention Follow-up Form was used to record the physiological parameters of the newborn before, during, and after the procedure.

#### The Neonatal Infant Pain Scale (NIPS)

This scale, developed by Lawrence et al. [29] in 1993 to evaluate the behavioral and physiological pain responses of preterm and term infants, was adapted to Turkish by Akdovan et al. [30] in 1999. The Cronbach alpha coefficient of consistency was 0.83 during the procedure. The Cronbach alpha was 0.75 in the treatment group and 0.88 in the control group during the vaccination procedure. The scale consists of one physiological section and five behavioral sections, including facial expression, cry, breathing pattern, arms and legs, and state of arousal. The cry section is scored between 0 and 2 points, and the other sections are scored between 0 and 1 point. The total score varies between 0 and 7 points, and a higher score indicates more pain [29, 30] (Table 1).

**Table 1:** Neonatal Infant Pain Scale (NIPS)

Parameters	0 point	1 point	2 point
Facial expression	Relaxed	Grimace	-
Cry	No cry	Whimper	Vigorous crying
Breathing pattern	Relaxed	Change in breathing	-
Arms	Relaxed	Flexed/extended	-
Legs	Relaxed	Flexed/extended	-
State of Arousal	Sleeping/Awake	Fussy	-

Pain level: 0–2 points = No pain, 3–4 points = Moderate pain, >4 points = Severe pain

### Procedure

All newborns received a hepatitis B vaccination during the study. Because different practitioners may cause different levels of pain perception, the newborns were vaccinated by the same nurse. Vaccination was performed on the vastus lateralis muscle. Physiological parameters were recorded by a nurse with a monitor starting from 15 s before the procedure. The procedure was recorded using a video camera. Video records were evaluated independently by four specialist observers (pediatric specialist nurse, neonatal doctor, pediatrician who was receiving a minor specialty education in neonate health, and a pediatric neurology specialist). Observers were not informed which newborn belonged to the control group and which newborn belonged to the treatment group. Observers scored the NIPS by evaluating the pain experienced by the newborns. The concordance coefficient was calculated between observers. A good level of concordance was found among the observers (Kappa = 0.65).

#### Treatment Group

**Fig. 2:**
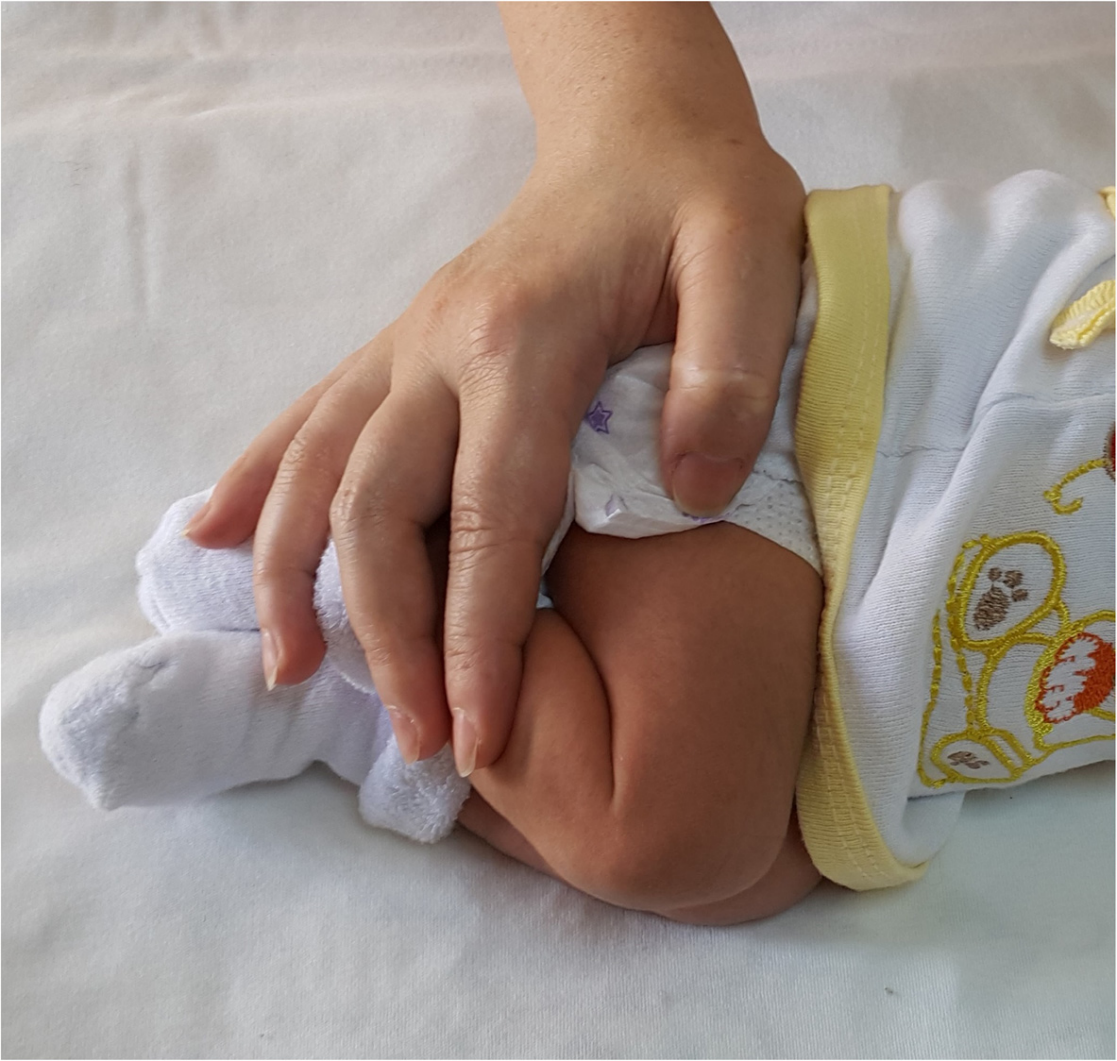
The infant in the facilitated tucking position

Each newborn in the treatment group was prepared in the facilitated tucking position 1 min before the procedure by the assistant nurse (Fig. 2), and 70 % alcohol was used to clean the area to be vaccinated in accordance with the clinical protocol. After the alcohol had evaporated, vaccination was performed by the nurse practitioner. Video recording was started 1 min before the vaccination and ended 1 min after. The physiological parameters measured by the bedside monitor (heart rate, body temperature, respiration, oxygen saturation) were recorded on the Intervention Follow-up Form before, during, and after the procedure.

#### Control Group

**Fig. 3:**
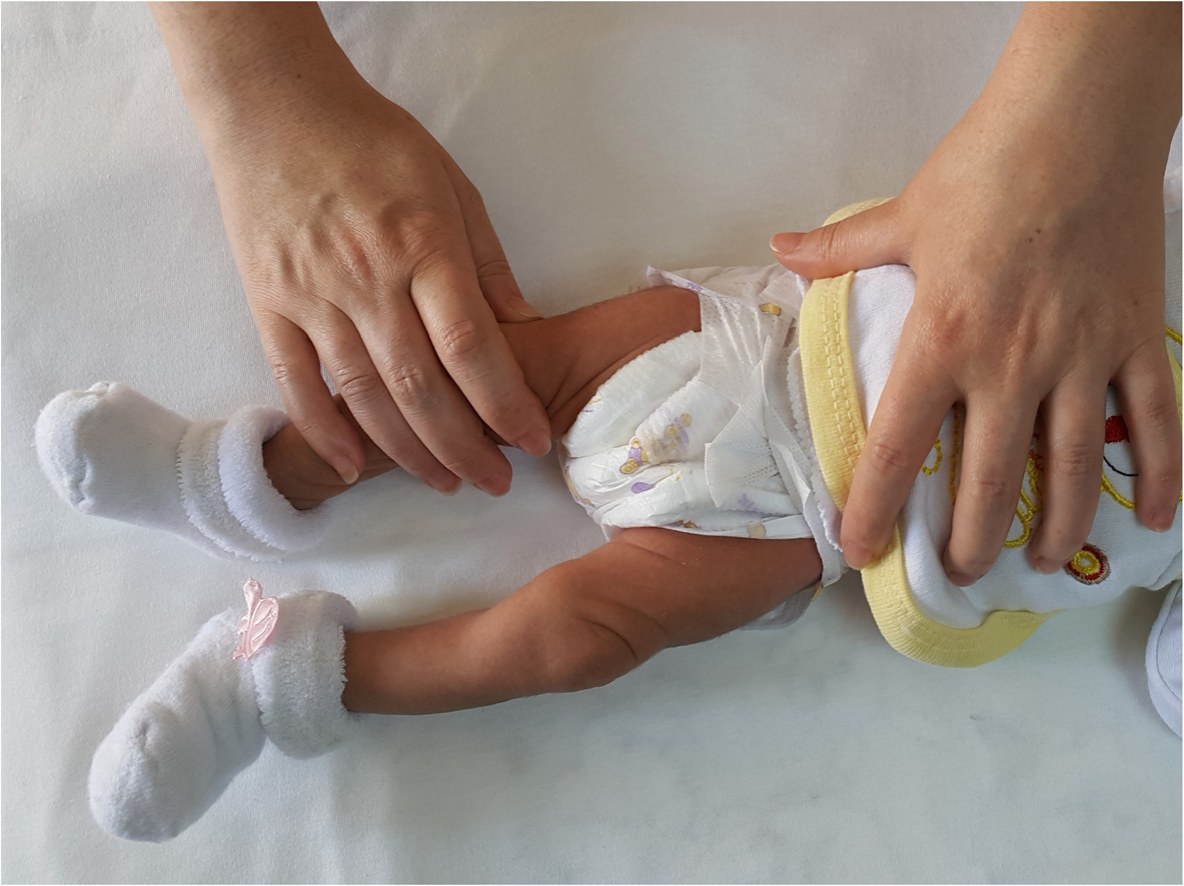
The infant in the classical holding position

No application was performed on the newborns in the control group. Vaccination was performed in the supine position on infants in this group as routine clinical practice. The leg that received the vaccination was brought to a straight position (classical holding position) (Fig. 3), and 70 % alcohol was used to clean the area to be vaccinated in accordance with the clinical protocol. After the alcohol had evaporated, the vaccination was performed by the nurse practitioner. Video recording was started 1 min before vaccination and ended 1 min after. The physiological parameters measured by the bedside monitor (heart rate, body temperature, respiration, oxygen saturation) were recorded on the Intervention Follow-up Form before, during, and after the procedure.

### Data analysis

The data were analyzed with the Statistical Package for Social Sciences (SPSS), version 20.0 (PASW ver. 20, SPSS Inc., Armonk, NY). Percentage distribution, mean, chi-square test, independent samples *t*-test, and Cronbach’s alpha coefficient calculation, as well as the kappa test for agreement analysis among independent observers, were used to assess the data. The results were assessed at a confidence interval of 95 % and with a significance level of *p* < 0.05.

### Ethical considerations

Approval was received from Atatürk University Faculty of Health Sciences Ethics Committee, and official permission from the hospital where the study was conducted was obtained. Additionally, informed written consent was obtained from each family included in the study.

## Results

Table 1 illustrates the comparison of the descriptive characteristics of the newborns in the control and treatment groups. There was no statistically significant difference between the groups in terms of gender, gestational age, weight, height, delivery method, and number of children previously delivered by the child’s mother (*p* > 0.05, Table 2). The mean NIPS pain scores of the infants in the treatment group (2.83 ± 1.18) were significantly lower than the scores of the infants in the control group (6.47 ± 1.07, *p* < 0.05). When 50 % of the infants in the treatment group had no pain, 93.4 % of the infants in the control group had severe pain. (Table 3). When the changes in the physiological parameters of newborns in the treatment and control groups were examined, no difference was found between the groups in terms of fever, pulse, respiration, and oxygen saturation before and after the procedure (*p* > 0.05, Table 4). The respiration rate of newborns in the control group was significantly higher compared to the newborns in the treatment group during the procedure (*p* < 0.05, Table 4).

**Table 2:** Comparison of control and treatment groups according to the newborn’s descriptive characteristics

Variables	Treatment group Mean ± SD	Control group Mean ± SD	Test and p
Gender*			
Female	19 (63.3)	15 (50.0)	*χ*2 = 1.086
Male	11 (36.7)	15 (50.0)	*p* = 0.217
Gestational age (week)	38.71 ± 0.75	38.86 ± 0.77	t = 0.733
			*p* = 0.467
Birth weight (g)	3318.00 ± 361.19	3380.33 ± 280.28	t = 0.855
			*p* = 0.396
Height (cm)	48.85 ± 2.15	49.13 ± 1.55	t = 0.585
			*p* = 0.561
Delivery method*			
Vaginal	2 (6.7)	4 (13.3)	*χ*2 = 0.71
Cesarean	28 (93.3)	26 (86.7)	*p* = 0.335
Number of delivery*			
Primipara	21 (70.0)	23 (76.7)	*χ*2 = 0.341
Multipara	9 (30.0)	7 (23.3)	*p* = 0.386

*n (%)

**Table 3:** Comparison of mean NIPS scores of newborns in the control and treatment groups

	Treatment group Mean ± SD	Control group Mean ± SD	Test and p
NIPS scores	2.83 ± 1.18	6.47 ± 1.07	t = 12.489
			*p* = 0.000
Pain level groups*			
0-2 points (No pain)	15 (50.0)	1 (3.3)	
3-4 points (Moderate pain)	11 (36.7)	1 (3.3)	
>4 points (Severe pain)	4 (13.3)	28 (93.4)	

*n (%)

**Table 4:** Comparison of the physiological parameters of the treatment and control groups before, during, and after the procedure

	Treatment group Mean ± SD	Control group Mean ± SD	Test and p
Before procedure			
Fever	36.62 ± 0.37	36.76 ± 0.32	t = 1.606 p = 0.114
Pulse	132.90 ± 16.09	140.30 ± 15.41	t = 1.819 p = 0.074
Respiration rate	47.70 ± 7.99	48.93 ± 9.90	t = 0.531 p = 0.598
SpO_2_^a^	96.60 ± 2.77	96.40 ± 3.01	t = 0.267 p = 0.790
During procedure			
Fever	36.79 ± 0.28	36.80 ± 0.30	t = 0.220 p = 0.826
Pulse	146.43 ± 28.61	156.23 ± 15.94	t = 1.639 p = 0.107
Respiration rate SpO_2_	44.83 ± 14.01	54.47 ± 13.59	t = 2.703 p = 0.009
SpO_2_	94.47 ± 4.22	94.37 ± 5.08	t = 0.083 p = 0.934
After procedure			
Fever	36.71 ± 0.30	36.80 ± 0.31	t = 1.440 p = 0.257
Pulse	141.23 ± 18.87	139.20 ± 17.97	t = 0.427 p = 0.427
Respiration rate	51.93 ± 11.12	53.33 ± 12.38	t = 0.461 p = 0.647
SpO2	95.97 ± 3.18	94.93 ± 3.47	t = 1.202 p = 0.234

^a^Oxygen saturation

## Discussion

According to the synactive theory, the facilitated tucking position is a non-pharmacological pain method that helps infants feel safe, preserve their energy, calm themselves, and reduce their oxygen consumption [15, 16]. During the classical holding position, which does not include a developmental care technique such as embracement and touching, the infant cannot feel safe. This causes the newborn to perceive more pain. The needs of each newborn should be considered by controlling and organizing environmental factors and applying care requirements to support each child’s development and life adaptation skills [31]. Controlling environmental stimuli also ensures that the newborn is calmed in addition to feeling pain relief [32]. The care plan should be monitored by using pharmacological and non-pharmacological methods to control the newborn’s pain [33].

Pain severity should be evaluated with multidimensional pain scales suitable for infant conditions and on which validity and reliability studies have been conducted to evaluate pain felt by newborns [34]. Individualized developmental care techniques and an environment that supports the newborn’s developmental abilities and enables the newborn to cope with stress and pain should be created [35]. In this study, infants were held in the facilitated tucking position during HBV vaccination, their pain perceptions were evaluated, and the pain perceptions of newborns held in this position were significantly lower compared to those held in the classical holding position. When examined the literature, facilitated-tucking position has been proven in many studies to be an effective method for relieving many procedural pain (such as heelstick, suctioning, venipuncture) in the infants [13, 15, 17–24]. However, any work that examined the impact of facilitated-tucking position relieving the pain occurred in hepatitis B and other vaccine applications has not been found. In a study conducted by Çağlayan [36] to analyze the effect of the facilitated tucking position, given by hand during the procedure to collect blood from the heel in preterm infants, infants in the facilitated tucking position felt less pain, similar to the results of this study. Ward-Larson et al. [13] performed endotracheal aspiration of 40 preterm infants in the facilitated tucking position and the routine position (in their own position), and the pain levels of babies in the facilitated tucking position were lower. Other studies have also reported that the facilitated tucking position is an effective method for relieving pain during painful procedures [12, 15–24]. Similar to the results of this study, the facilitated tucking position was effective in relieving procedural pain in Lopez et al.’s study [18].

Physiological changes may be part of a newborn’s response to pain; these changes should be monitored until the parameters return to their normal values during the procedure [10]. Physiological symptoms caused by painful stimulators indicate the general stress state of the body. Although the most common physiological symptoms such as heart rate, blood pressure, respiration, and oxygen saturation are used to evaluate pain occurring due to acute procedures, hormonal and metabolic variables are also used to assess pain [32]. No statistical difference was observed between the groups in terms of the pre-procedural and post-procedural respiration, heart rate, fever, and SpO_2_ mean scores, a significant difference was found between the groups in terms of respiration during the procedure. Normal respiration values were between 30 and 60/min in the physical evaluation of newborns [37]. In this study, the respiration rates of the infants in the control group were within normal limits although the rates were higher than the rates of the infants in the treatment group.

## Conclusions

The facilitated tucking position was more effective than the routine position in relieving pain that occurred due to vaccination. Therefore, this position can be used in conjunction with pharmacological methods during painful procedures due to its simple, inexpensive, and non-invasive application. Additional evidence-based studies that use facilitated tucking in other areas of newborn care should be conducted.

## References

[1] Kyle T, Carman S: Essentials of Pediatric Nursing: Pain Management in Children. 2nd Edition. Philadelphia, PA: Lippincott Williams & Wilkins.2012.

[2] Tekin NE, Hasanoğlu R, Düşünsel AB (2010). Pain in Newborn: Basic Pediatrics.

[3] Walden M, Verklan MT, Walden M (2010). Pain assessment and management. Core curriculum for Neonatal Intensive Care Nursing.

[4] Reis EC, Roth EK, Syphan JL, Tarbell SE, Holubkov R (2003). Effective pain reduction for multiple immunization injections in young infants. Arch Pediatr Adolesc Med.

[5] Young KD (2005). Pediatric procedural pain. Ann Emerg Med.

[6] Gradin M, Eriksson M, Holmqvist G, Holstein A, Schollin J (2002). Pain reduction at venipuncture in newborns: oral glucose compared with local anesthetic cream. Pediatrics.

[7] Ommaty R (2009). Vademacum: Vital Pharmy Index. Ankara.

[8] Faye MP, Jonckheere JD, Logier R, Kuissi E, Jeanne M, Rakza T (2010). Newborn infant pain assessment using hearth rate variability analysis. Clin J Pain.

[9] Anand KJS, Hall RW, Desai N, Shephard B, Bergqvist LL, Young TE (2004). Effects of morphine analgesia in ventilated preterm neonates: primary outcomes from the NEOPAIN randomised trial. Lancet.

[10] Lago P, Garetti E, Merazzi D, Pieragostini L, Ancora G, Pirella A (2009). Guidelines for procedural pain in the newborn. Acta Paediatr.

[11] Ludington-Hoe SM, Hosseini RB (2005). Skin-to-skin contact analgesia for preterm infant hell stick. AACN Clin Issues.

[12] Obeidat H, Kahalaf I, Callister LC, Froelicher ES (2009). Use of facilitated tucking for nonpharmacological pain managment in preterm infants: A systematic review. J Perinat Neonatal Nurs.

[13] Ward-Larson C, Horn RA, Gosnell F (2004). The efficacy of facilitated tucking for relieving procedural pain of endotracheal suctioning in very low birthweight infants. MCN Am J Matern Child Nurs.

[14] Huang CM, Tung WS, Kuo LL, Chang YJ (2004). Comparison of pain responses of premature infants to the heelstick between containment and swaddling. J Nurs Res.

[15] Axelin A, Salanterä S, Lehtonen L (2006). “Facilitated tucking by parents” in pain management of preterm infants: a randomized crossover trial. Early Hum Dev.

[16] Hill S, Engle S, Jorgensen J, Kralik A, Whitman K (2005). Effects of facilitated tucking during routine care of infants born preterm. Pediatr Phys Ther.

[17] American Academy of Pediatrics, Committee on Fetus and Newborn, Canadian Paediatric Society, Fetus and Newborn Committee (2007). Prevention and management of pain in the neonate: an update. Adv Neonatal Care.

[18] Lopez O, Subramanian P, Rahmat N, Chin Theam L, Chinna K, Rosli R (2015). The effect of facilitated tucking on procedural pain control among premature babies. J Clin Nurs.

[19] Liaw JJ, Yang L, Katherine Wang KW, Chen CM, Chang YC, Yin T (2012). Non-nutritive sucking and facilitated tucking relieve preterm infant pain during heel-stick procedures: a prospective, randomized controlled crossover trial. Int J Nurs Stud.

[20] Çağlayan N, Balcı S (2014). An effective model of reducing pain in preterm neonates: facilitated tucking. FN Hem Derg.

[21] Cignacco EL, Sellam G, Stoffel L, Gerull R, Nelle M, Anand KJ (2012). Oral sucrose and “facilitated tucking” for repeated pain relief in preterms: a randomized controlled trial. Pediatrics.

[22] Hartley KA, Miller CS, Gephart SM (2015). Facilitated tucking to reduce pain in neonates: evidence for best practice. Adv Neonatal Care.

[23] Peyrovi H, Alinejad-Naenini M, Mohagheghi P, Mehran A (2014). The effect of facilitated tucking position during endotracheal suctioning on physiological responses and coping with stress in premature infants: a randomized controlled crossover study. J Matern Fetal Neonatal Med.

[24] Alinejad-Naenini M, Mohagheghi P, Peyrovi H, Mehran A (2014). The effect of facilitated tucking during endotracheal suctioning on procedural pain in preterm neonates: a randomized controlled crossover study. Glob J Health Sci.

[25] Vessey JA, Carlson KL, McGill J (1994). Use of distraction with children during an acute pain experience. Nurs Res.

[26] Karaayvaz T (2009). The effectiveness of oral sucrose and topical EMLA application in reducing neonatal pain during venipuncture.

[27] Axelin A. Parents as pain killers in the pain management of preterm infants. 2010. http://www.doria.fi/bitstream/handle/10024/63939/AnnalesD916.pdf?sequence. Accessed 28 December 2014.

[28] Schulz KF, Grimes DA (2002). Generation of allocation sequences in randomized trials: chance, not choice. Lancet.

[29] Lawrence J, Alcock D, McGrath P, Kay J, MacMurray SB, Dulberg C (1993). The development of a tool to assess neonatal pain. Neonatal Netw.

[30] Akdovan T, Yıldırım Z (1999). Assessment of pain in healthy neonates, investigation of the effects of pacifying and holding in arms. Perinatal J.

[31] Als H, Lawhorn G, Kenner C, McGrath JM (2004). Theoretical perspective for developmentally supportive care. Developmental care of newborns and infants: A guide for health professionals. Glenview.

[32] Derebent E, Yiğit R (2006). Pain in newborn: assessment and management. CU Hemşirelik Yüksek Okulu Derg.

[33] Gardner SL, Hagedorn MIE, Dickey LA, Merenstein BG, Gardner SL (2006). Pain and Pain Relief. Handbook of Neonatal Intensive Care.

[34] Akyürek B, Conk Z (2006). The efficacy of non-pharmacological pain relief methods in injection: application to newborns. Ege Üniversitesi Hemşirelik Yüksek Okulu Dergisi.

[35] Dağoğlu T, Dağoğlu T, Görak G (2008). Newborn development and enviromental factors. Basic Neonatology and Nursing Principles.

[36] Çağlayan N (2011). The effect on pain of manipulating the preterm neonate into the facilitated tucking during drawing of blood from the heel.

[37] Karabudak S, Ergün S, Conk Z, Başbakkal Z, Yılmaz HB, Bolışık B (2013). Neonatal Diseases and Nursing Care. Pediatric Nursing.

